# Development of a Layer-by-Layer Zein/CMCS Microcapsule Platform for Bacteriophage Delivery: A Proof-of-Concept Study Using a Model Phage in Sea Bass

**DOI:** 10.3390/foods15061032

**Published:** 2026-03-16

**Authors:** Weiquan Liang, Tangwu Qiu, Zheng Cheng, Yunqian Sun, Yunyun Zhong, Xueqin Zhang, Le Zhong

**Affiliations:** 1 College of Light Industry and Food Technology, Zhongkai University of Agriculture and Engineering, Guangzhou 510225, China; 18378386847@163.com (W.L.); 15767379563@163.com (T.Q.); chengzheng@zhku.edu.cn (Z.C.); syqqq0129@126.com (Y.S.); zhongyunyun@zhku.edu.cn (Y.Z.); zhangxueqin0228@163.com (X.Z.); 2 Guangdong Engineering Technology Research Center for Food Green Packaging, Zhongkai University of Agriculture and Engineering, Guangzhou 510225, China; 3 Key Laboratory of Green Processing and Intelligent Manufacturing of Lingnan Specialty Food, Ministry of Agriculture and Rural Affairs, Zhongkai University of Agriculture and Engineering, Guangzhou 510225, China

**Keywords:** bacteriophage, layer-by-layer, microcapsule, fresh-cut sea bass, biopreservation

## Abstract

Bacteriophages (phages) offer a targeted biocontrol solution, but their direct application is hampered by environmental instability. To address this, we developed a novel, food-grade microcapsule system for phage delivery using layer-by-layer (LbL) self-assembly of zein and carboxymethyl chitosan (CMCS). Lytic phages targeting specific spoilage bacteria were successfully encapsulated via electrostatic interactions. Characterization confirmed the formation of a multilayer structure, driven primarily by hydrogen bonding and electrostatic forces between the wall materials. The microencapsulation markedly enhanced phage stability against thermal (60 °C and 70 °C) and extreme pH (2.0, 12.0) stresses and provided a controlled release profile in a simulated fish exudate. When applied to fresh-cut sea bass (*Lateolabrax japonicus*), the phage-loaded microcapsules (CMCS3), constructed via a three-layer zein/CMCS LbL assembly, significantly delayed the pH rise during refrigerated storage, maintaining a final pH of 6.28 compared to 7.28 in the control group after 5 days. The microcapsules also effectively suppressed microbial growth (total viable count (TVC) was maintained below 6 log CFU/g) and controlled lipid oxidation (thiobarbituric acid reactive substances (TBARS) values were kept at 0.62 mg malondialdehyde/kg) while better preserving texture and color stability compared to free phages. This zein/CMCS-based LbL system presents a promising strategy for advancing phage-based biopreservation in aquatic products through enhanced physical protection, sustained release, and improved stress tolerance.

## 1. Introduction

Fresh-cut fish products, such as sea bass (*Lateolabrax japonicus*) slices, are highly perishable due to their high moisture content and nutrient-rich matrix, which favors the rapid proliferation of spoilage microorganisms [1,2], coupled with endogenous enzymes that cause the hydrolysis of muscle proteins and the oxidation of lipids [3]. Traditional preservation strategies—such as refrigeration, modified atmosphere packaging (MAP), and chemical preservatives—are often limited by their short-lived efficacy, potential to induce undesirable flavor changes, or concerns regarding chemical residues [4,5]. Consequently, there is a growing demand for natural and safe preservation alternatives.

Bacteriophages (phages), as a type of virus capable of specifically lysing host bacteria, are promising natural targeted biological control agents [6]. Their core advantages lie in high host specificity [7], reliable safety, and no adverse impact on the sensory quality of food; they have been recognized as GRAS (generally recognized as safe) by regulatory bodies such as the FDA [8]. However, there are still obstacles to the direct application of bacteriophages in aquatic products, and the main challenge lies in environmental instability (pH [9], temperature fluctuations [10] and ionic strength, endemic proteases, exposure to carbohydrates [11]). All of them may induce the inactivation of phage particles, resulting in a significant decrease in the effective titer and ultimately restricting their practical application effects. Therefore, developing a delivery system that can protect bacteriophages and maintain their long-term activity has become the key to promoting their industrial application. So far, many phage coating techniques have been reported, and phages are surrounded in different forms, including liposomes [12], microspheres [13], nanofibers [14], and film [15].

Layer-by-layer (LbL) is a technique that deposits bioactive molecules by constructing ordered multilayer structures layer-by-layer through various intermolecular interactions, such as electrostatic interactions, hydrogen bonds, and van der Waals forces [16]. LbL has been widely applied in the packaging and delivery of environmentally sensitive components due to its advantages, such as adjustable process design, multi-functional templates, simple and gentle operating conditions, and natural adsorption drive [17]. Given the above-mentioned advantages of LbL technology, it is highly suitable for encapsulating and protecting environmentally sensitive bioactive substances such as bacteriophages. Therefore, to achieve effective LbL encapsulation, the key is to select the appropriate wall material.

Zein and carboxymethyl chitosan (CMCS) were selected for the LbL system based on structural, functional, and safety considerations. CMCS, an anionic polysaccharide, was able to adsorb onto bacteriophage surfaces via electrostatic interactions to form stable primary complexes. It further interacted firmly with positively charged polyelectrolytes to produce stable bilayer and multilayer shells through LbL assembly. Additionally, CMCS exhibited good biocompatibility, mucoadhesive properties, and inherent antibacterial activity [18], which could synergistically enhance preservation. Zein was chosen as an LbL substrate due to its excellent film-forming ability, biocompatibility, biodegradability, self-assembly characteristics [19], and amphiphilic nature. Its unique hydrophobicity and amphiphilic structure enabled its use in delivery systems for various functional compounds, including fat-soluble vitamins [20], polyphenols [21,22], essential oils [23,24], and probiotics [25,26]. Importantly, zein are classified as generally recognized as safe (GRAS) [27], ensuring full food-grade safety—a decisive advantage over many synthetic or non-GRAS alternatives.

**Figure 1:**
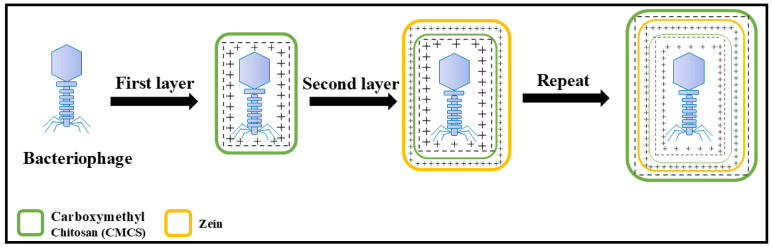
Diagram of phage encapsulation by layer-by-layer assembly method. The “+” and “−” symbols denote positive and negative surface charges, respectively.

However, studies on the comprehensive utilization of these two food-grade materials (zein and CMCS) and the design of LbL sequences based on their electrostatic complementarity to encapsulate bacteriophages have not been reported so far. Therefore, the aim of this study was to develop and design a new phage microcapsule by LbL technology using CMCS and zein as wall materials. A schematic representation of the phage encapsulation process via LbL assembly is illustrated in Figure 1. In this study, microcapsules based on zein were prepared by the anti-solvent precipitation method, and the commonly used anionic polysaccharide carboxymethyl chitosan was selected as another coating unit. The interaction mechanism between zein and CMCS was analyzed by zeta potential, sodium dodecyl sulfate-polyacrylamide gel electrophoresis (SDS-PAGE) and Fourier transform infrared spectroscopy (FT-IR), and the stable protective effect of layer-by-layer self-assembled microcapsules as coating layers on phages under pH, heating and storage conditions was evaluated. Additionally, a preservation experiment was conducted on sea bass using a simplified model to preliminarily compare the effects of the phage-loaded microcapsules with free phages.

## 2. Materials and Methods

### 2.1. Materials

Host strain: The host strain FB06, identified as *Acinetobacter junii* via 16S rRNA gene sequencing, was previously isolated from spoiled sea bass. The 16S rRNA gene sequencing data for the bacterial strains used in this study have been deposited and are available as Supplementary Materials. The bacterial strain was maintained long-term in LB broth containing 40% glycerol at −80 °C.

Bacteriophages: A lytic bacteriophage (named Zk-X6) targeting FB06 was isolated from fish market wastewater and propagated in LB broth.

Carboxymethyl chitosan and zein,1,1,3,3-tetraethoxypropane (TEP) were purchased from Macklin Biochemical Technology Co., Ltd. (Shanghai, China) and Yuanye Bio-Technology Co., Ltd. (Shanghai, China), respectively. All other chemical reagents, including nutritious broth, were of analytical grade and obtained from commercial suppliers. Deionized water was used throughout the experiments.

### 2.2. Methods

#### 2.2.1. Preparation of Zein Nanodispersion

The method of Liu Bu [28] was referred to and slightly modified. Briefly, 1.0 g of zein powder was dissolved in 50 mL of 80% (*v*/*v*) ethanol solution at room temperature (25 ± 1 °C) with magnetic stirring at 500 rpm for 30 min, followed by standing for 24 h to ensure complete dissolution. Then, under constant-temperature water bath conditions at 25 ± 1 °C, the above zein–ethanol solution was slowly injected into 450 mL of continuously stirred (500 rpm) deionized water at a rate of 1 mL/s to induce protein anti-solvent precipitation and form nanoparticles. After stirring for another 10 min, the solution was treated with a rotary evaporator (45 °C) for 2 h to completely remove the residual ethanol. Finally, the pH of the dispersion was adjusted to 5.0 with 0.1 M HCl or NaOH solution and stored at 4 °C in a refrigerator for future use. The final concentration of the obtained zein nanoparticles aqueous dispersion was 2 mg/mL.

#### 2.2.2. Preparation of CMCS Solution

Accurately weigh 400 mg of carboxymethyl chitosan powder and dissolve it in 200 mL of ultrapure water. Stir magnetically until completely dissolved. The pH of the obtained CMCS solution was adjusted to 5.0 using 0.1 M HCl or NaOH solution to obtain a stock solution with a concentration of 2 mg/mL, which was kept in a 4 °C refrigerator for future use.

#### 2.2.3. Preparation of CMCS–Phage Microcapsules

1. Deposition of the first layer (CMCS1): A total of 9 mL of CMCS solution (2 mg/mL, pH 5.0) was mixed with 1 mL of phage suspension in a 15 mL centrifuge tube. The mixture was gently vortexed at room temperature (25 °C) and then allowed to stand for 15 min to enable the adsorption of negatively charged CMCS onto the positively charged phage surface. Subsequently, it was centrifuged at 4000× *g* for 10 min at 4 °C, and the supernatant was discarded to remove unbound CMCS. The resulting precipitate was resuspended in 10 mL of sterile phosphate-buffered saline (PBS) and washed twice via centrifugation. The final product was designated as CMCS1.

2. Deposition of the second layer (CMCS2): The CMCS1 precipitate was resuspended in 10 mL of zein nanodispersion (2 mg/mL, pH 5.0) and gently stirred at room temperature for 15 min to allow the adsorption of positively charged zein onto the negatively charged surface of CMCS1. Centrifugation was performed under the same conditions (4000× *g*, 4 °C, 10 min), the supernatant was discarded, and the pellet was washed twice with PBS. The resulting bilayer microcapsules were designated as CMCS2.

3. Deposition of the third layer (CMCS3): To construct the third layer, the CMCS2 precipitate was resuspended in 10 mL of fresh CMCS solution (2 mg/mL, pH 5.0). The adsorption, centrifugation, and washing procedures described in step (1) were repeated. Finally, microcapsules with three layers were obtained and denoted as CMCS3.

### 2.3. Viability of Phages During LbL Assembly

The encapsulation efficiency (*EE*) of the LbL process was calculated to evaluate the retention of phage activity throughout the assembly steps. It was determined by comparing the titer of phages recovered from the ruptured microcapsules to the titer of the initial phage suspension used for encapsulation:

(1) $$EE=\frac{N_{encapsulated}}{N_{initial}}\times 100\%$$

where *EE* is the encapsulation efficiency (%). *N_encapsulated_* and *N_initial_* are the phage titers expressed in PFU/mL (plaque-forming units per milliliter). These values were obtained by converting the mean logarithmic titers (log PFU/mL) reported in Table 1 to their corresponding linear concentrations.

### 2.4. Zeta Potential Measurement

The zeta potential values of the uncoated bacteriophage and the three microcapsule formulations (CMCS1, CMCS2, CMCS3) were quantified at 25 °C using a NanoBrook 90 Plus PALS analyzer (Brookhaven Instruments, Nashua, NH, USA). Prior to measurement, each sample was appropriately diluted with ultrapure water (pH 5.0) to achieve a suitable scattering intensity. Each reported value represents the mean ± standard deviation of three independent measurements.

### 2.5. Sodium Dodecyl Sulfate-Polyacrylamide Gel Electrophoresis (SDS-PAGE)

Non-reducing SDS-PAGE was carried out by using a 4–20% precast gel. Aliquots of (10 μL, 2 mg/mL protein concentration) zein and microcapsules with varying layer numbers were mixed with 5 × loading buffer and then heated at 100 °C in a metal bath for 5 min. A total of 10 μL of each sample was loaded into the wells of the gel. The voltage was set at 160 V throughout electrophoresis, which lasted approximately 1 h. The gel was stained for 60 min using Coomassie brilliant blue G-250 solution as the staining medium. Subsequently, the gel was removed from a decolorizing bath containing Coomassie Brilliant Blue decolorizing solution for 60 min, with the decolorizing solution replaced every 20 min. Finally, the electrophoresis image was recorded.

### 2.6. Fourier Transform-Infrared Spectroscopy (FT-IR) Analysis

The freeze-dried powders of microcapsules with different layers, zein, and CMCS were separately mixed with potassium bromide at a mass ratio of 1:100, thoroughly ground, and pressed into KBr pellets. The chemical structure and interactions of the microcapsules were analyzed using an FT-IR spectrometer (Nicolet 5700, Thermo Fisher Scientific, Waltham, MA, USA). Spectral signals were recorded in the wavenumber range of 400–4000 cm^−1^, with an average of 32 scans accumulated at a resolution of 1 cm^−1^.

### 2.7. Thermal Stability

To compare the thermal stability of microencapsulated bacteriophages and free bacteriophages, wet microcapsules or 1 mL of bacteriophage suspension was added to sterile tubes, followed by 9 mL of sterile deionized water. The tubes were then incubated at 60 °C and 70 °C for 60 min, respectively. After heating was completed, 10 mL of SuperMicro buffer was added for rapid cooling, and then, the surviving bacteriophages were counted.

### 2.8. pH Treatment

To compare the pH stability of microencapsulated phage and free phages, wet phage microcapsules or 1 mL phage suspension was added into the sterile tube, and 9 mL sterile deionized water was added and kept at pH 2.0 or pH 12.0 for 60 min, respectively. By serially diluting in sterile deionized water, the titers of phages were determined using the double-agar overlay plaque assays.

### 2.9. The Stability of Microencapsulated Bacteriophages on Sea Bass Slices

A total of 5 g of sterile sea bass slices was immersed in an FB06 bacterial solution with a concentration of 1.0 × 10^6^ CFU/mL for 2 min and then dried in a sterile environment for 10 min. Then, it was immersed in one 20 mL free bacteriophage or one 20 mL CMCS3 suspension, drained, vacuumed in a sterile bag, and stored at 4 °C. The bacteriophage titers were measured at 0 and 5 days.

### 2.10. The Release Behavior of Bacteriophages Microcapsules Storage

The in vitro release profile was investigated under accelerated conditions (37 °C with agitation at 220 rpm). This temperature was selected to enable a clear comparative analysis of the release kinetics among different microcapsule formulations (CMCS1, CMCS2, CMCS3) within a practical experimental timeframe. Elevated temperature accelerates molecular diffusion and polymer relaxation, thereby facilitating the observation of complete release trends. Accordingly, this assay was primarily employed to compare the relative release profiles and structural integrity of the formulations, rather than to precisely simulate the absolute release rate under actual refrigerated storage.

Briefly, wet microcapsules or 1 mL of free phage suspension (control) was placed into a tube containing 9 mL of simulated fish exudate (composed of 8 mL of PBS and 1 mL of 0.6% sodium citrate solution, prepared according to a modified method [29]). After thorough mixing, the tubes were incubated in a shaking incubator at 37 °C and 220 rpm. Aliquots (100 μL) were collected at predetermined time intervals (2, 4, 6, and 8 h). The phage titer in each aliquot was immediately determined using the standard double-agar overlay plaque assay.

### 2.11. pH Determination of Sea Bass Slices During Refrigeration Storage

For pH determination, 1 g of homogenized fish sample was extracted with 10 mL of potassium chloride (0.1 M) solution in a 50 mL centrifuge tube. After centrifugation at 8000 rpm for 10 min, the pH of the supernatant was measured with a calibrated pH meter (PHSJ-3C, INESA, Shanghai, China).

### 2.12. Total Viable Count (TVC) Determination of Sea Bass Slices During Refrigeration Storage

The fish sample was placed on an ultra-clean worktable; a total of 2.5 g of the sample was weighed into a sterilized homogenization bag containing 22.5 mL of 0.85% sterile normal saline. After sealing, the bag was patted for 2 min using a homogenizer. The homogenate was transferred to a sterile centrifuge tube, vortexed and mixed well, and then subjected to a 10-fold serial dilution. From the appropriate dilutions, 1 mL was suctioned into a sterilized dish, which was followed by adding approximately 20 mL of sterilized plate counting agar medium and shaking well. After the agar solidified, the plate was inverted and incubated at 30 °C for 24 h. Finally, the colonies were counted, and the results were expressed as the logarithm of colony-forming units per gram (CFU/g).

### 2.13. Thiobarbituric Acid Reactive Substances (TBARS) Determination of Sea Bass Slices During Refrigeration Storage

The TBARS content was determined as malondialdehyde (MDA) equivalents, following a modified procedure based on GB 5009.181-2016 [30]. Briefly, 5.0 g of minced sea bass was homogenized with 50 mL of a trichloroacetic acid (TCA) mixture containing 7.5% TCA and 0.1% EDTA-2Na. The homogenate was vortexed for 2 min, followed by orbital shaking for 30 min. After centrifugation and filtration, 5 mL of the clear filtrate was reacted with 5 mL of 0.02 mol/L thiobarbituric acid (TBA) solution at 90 °C for 30 min. The mixture was cooled, and the absorbance of the resulting chromogen was measured at 532 nm. For quantification, a standard curve was constructed using TEP at concentrations of 0.01, 0.05, 0.10, 0.15, and 0.25 μg/mL (as MDA equivalents). The TEP standards underwent the same color development procedure as the samples. The TBARS content of the samples was calculated based on the linear regression equation derived from this standard curve and expressed as milligrams of MDA per kilogram of sample (mg MDA/kg).

### 2.14. Total Volatile Basic Nitrogen (TVB-N) Determination of Sea Bass Slices During Refrigeration Storage

Five grams of the minced fish sample was placed in a distillation tube, followed by the addition of 37.5 mL of deionized water. After mixing well, the mixture was allowed to soak for 30 min. Subsequently, 1 g of magnesium oxide was added, and the TVB-N content was determined using an automatic Kjeldahl nitrogen analyzer (Hai Neng K9860, Jinan, China). The results were expressed as milligrams of volatile basic nitrogen per 100 g of sample.

### 2.15. Color Determination of Sea Bass Slices During Refrigeration Storage

The color of sea bass slices was measured using a colorimeter. Prior to measurement, the instrument was calibrated with standard white and black calibration tiles. For each sample, three points were randomly selected on the surface of the fish slice, avoiding visible blood vessels and fascia. The measurement window was cleaned with lens paper before each reading. The values of L* (lightness), a* (redness), and b* (yellowness) were recorded. The total color difference (ΔE) was then calculated from the measured values using the following formula:

(2) $$\Delta E=\sqrt{{\left(\Delta L*\right)}^{2}+{\left(\Delta a*\right)}^{2}+{\left(\Delta b*\right)}^{2}}$$

### 2.16. Statistical Analysis

All data are expressed as the mean ± standard deviation of three independent replicates. Microbial counts were log10-transformed prior to statistical analysis. Data were analyzed using SPSS Statistics 24.0. A one-way analysis of variance (ANOVA) was first performed to assess overall differences among treatment groups. When ANOVA indicated a significant difference (*p* < 0.05), post hoc multiple comparisons were conducted using Tukey’s honestly significant difference (HSD) test to evaluate pairwise differences. The significance level was set at α = 0.05. For the presentation of results, data in figures and tables are shown as mean ± SD (*n* = 3). Within the same storage time point, mean values bearing different superscript letters indicate statistically significant differences between treatment groups (*p* < 0.05), whereas the same letter denotes no significant difference.

## 3. Results

### 3.1. Effect of LbL Assembly Process on the Phage Survival

The influence of microcapsules with three encapsulation layers on the phage titer is shown in Table 1. The phage activity before and after coating was characterized for microcapsules with different numbers of layers, and it was found that the titer of the one-layer (CMCS1) phage microcapsule group decreased from 5.14 ± 0.01 to 4.05 ± 0.04 log PFU/mL. The titer of the two-layer (CMCS2) phage microcapsule group decreased from 5.14 ± 0.01 to 4 ± 0.07 log PFU/mL, and that of the three-layer (CMCS3) phage microcapsule group decreased from 5.14 ± 0.01 to 3.94 ± 0.06 log PFU/mL. Based on these logarithmic titers, the corresponding encapsulation efficiency (*EE*) was calculated using linear phage concentrations (PFU/mL) according to Equation (1). The *EE* values were 8.13%, 7.25%, and 6.31% for CMCS1, CMCS2, and CMCS3, respectively. They decreased by approximately 1.092 log PFU/mL, 1.136 log PFU/mL, and 1.204 log PFU/mL, respectively. The phage titers of the three types of microcapsules all decreased, which might be due to the inevitable phage loss caused by operations such as centrifugation during assembly, solution loading and transfer, and capsule rupture in high-speed dispersers. A similar situation also occurred in the related research on embedding probiotics. After Cigdem Falco et al. coated Lactobacillus acidophilus with two layers, the number of probiotics decreased by approximately 1 log CFU/mL (from 8.4 ± 0.1 to 7.2 ± 0.4) [31]. This parallel highlighted that procedural stresses inherent to the LbL assembly process can lead to a quantifiable decrease in the payload, regardless of the biological entity being encapsulated. It is worth noting that, during the process of breaking the microcapsules of each layer for potency determination, there may be cases where the microcapsules are not completely disintegrated. As a result, the actual measured live phage titers may be smaller, and there may be certain errors.

### 3.2. Change in Zeta Potential of Phage Microcapsules After LbL Assembly

**Figure 2:**
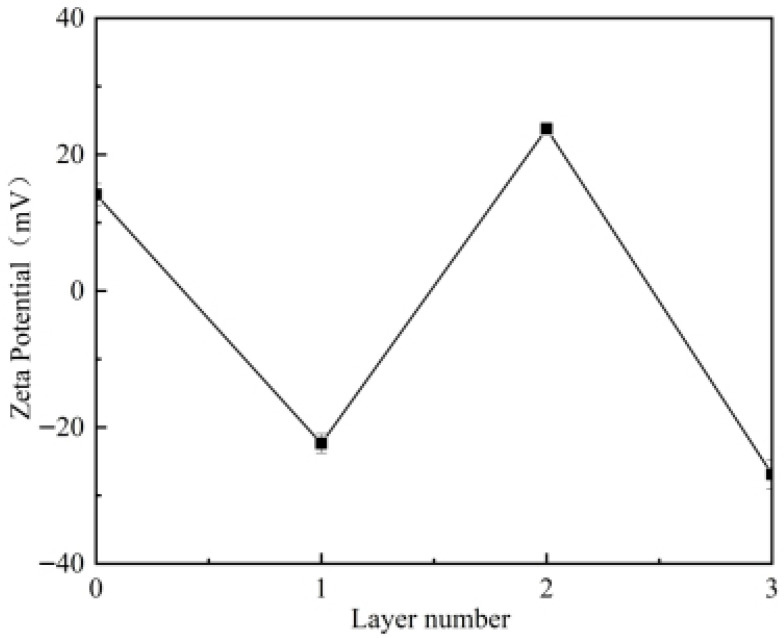
Zeta potential changes of the surface of phage and phage microcapsules coated with a different number of layers. Error bars represent the standard deviation (SD) of three independent replicates.

The anionic polymer CMCS was selected as the initial layer to deposit onto the positively charged phage surface via electrostatic attraction. Upon deposition, the surface potential reversed to −22.37 mV for the CMCS1 microcapsules. As shown in Figure 2, this alternating positive–negative pattern persisted through subsequent layering with zein and CMCS. This characteristic zeta potential reversal is a hallmark of successful LbL assembly and strongly suggests that electrostatic interaction is the primary driving force for the stepwise adsorption. In such bio-polyelectrolyte systems, secondary non-covalent interactions, such as hydrogen bonding, likely play a synergistic role in stabilizing the multilayered structure.

### 3.3. Characteristic of SDS of Phage Microcapsules After LbL Assembly

**Figure 3:**
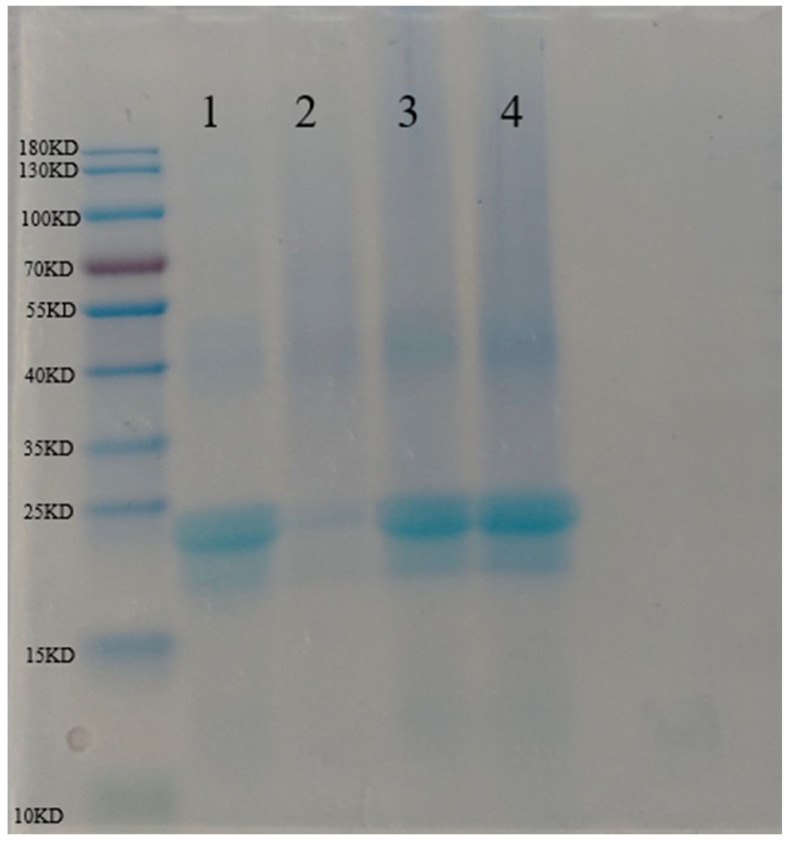
Sodium dodecyl sulfate-polyacrylamide gel electrophoresis (SDS-PAGE) of zein nanoparticles and phage microcapsules. Lane 1—zein nanoparticles, Lane 2—CMCS1, Lane 3—CMCS2, Lane 4—CMCS3.

Sodium dodecyl sulfate-polyacrylamide gel electrophoresis (SDS-PAGE) is a classic method for analyzing the expression of protein subunits and molecular weight changes; to explore the interaction force between bacteriophages and LbL substrates, electrophoresis experiments were performed (Figure 3). Characteristic bands of α-zein (22 KDa and 24 KDa) were observed in the ZNP profile (Lane 1), which were the same as the results of past studies [32]. The several microcapsules that were assembled did not produce new bands, which indicates that no covalent interaction was involved between the bacteriophage and the LbL substrate.

### 3.4. Characteristic of FT-IR of Phage Microcapsules After LbL Assembly

**Figure 4:**
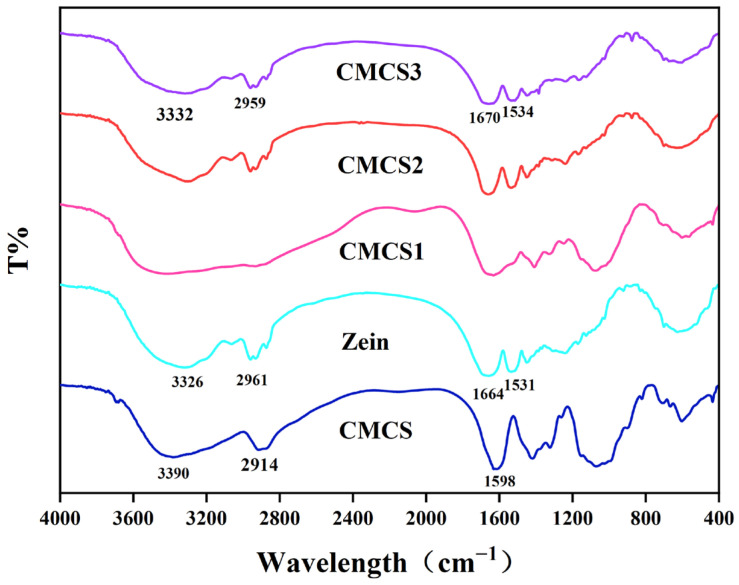
Fourier transform-infrared spectroscopy (FT-IR) spectra for zein, carboxymethyl chitosan (CMCS) and phage microcapsules with a different number of layers.

Fourier transform-infrared spectroscopy (FT-IR) analysis was performed to investigate the interactions between materials and identify characteristic functional groups (Figure 4). The FTIR spectrum of pure zein exhibited characteristic peaks at 3326, 2961, 1664 and 1531 cm^−1^, which are attributed to O–H stretching, C–H stretching, the C=O stretching of amide I, and the N–H bending of amide II, respectively [33]. For CMCS, the peak at 3390 cm^−1^ is assigned to the O–H stretch. The peak at 2914 cm^−1^ is related to a strong vibration of hydrophobic C–H stretching. The peak at 1598 cm^−1^ is ascribed to N–H bend [34]. Upon formation of the CMCS3 composite, the O–H stretching vibration shifted from 3326 cm^−1^ to 3332 cm^−1^, suggesting the formation of hydrogen bonds between zein and CMCS [35]. The observed amide I band of corn shifted from 1663 cm^−1^ to 1660 cm^−1^, suggesting that an electrostatic interaction occurred between zein and CMCS, which is consistent with the established literature [36].

### 3.5. Analysis of Phage Microcapsules Thermal Stability

**Figure 5:**
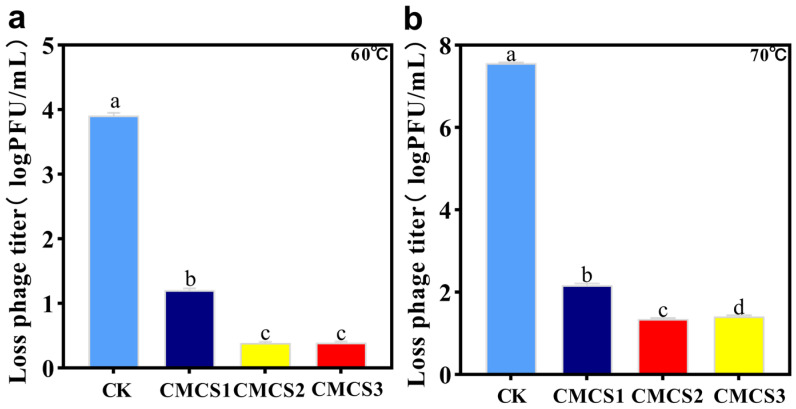
Titer loss of free phage and phage microcapsules with different layers under high-temperature conditions. (**a**) 60 °C; (**b**) 70 °C. Error bars represent the standard deviation (SD) of three independent replicates.

In practical applications, high temperature is a major constraint on the use of free bacteriophages, as the proteins in their heads are prone to denaturation, leading to the loss of enzyme activity and a decline in antibacterial ability. To improve its thermal stability, microencapsulation was employed in this study. After heating at 60 °C for 1 h, the titer of free phages decreased by 3.91 ± 0.04 log PFU/mL, while the titers of phages coated with CMCS1, CMCS2, and CMCS3 layers only decreased by 1.2 ± 0.03, 0.39 ± 0.02, and 0.39 ± 0.02 log PFU/mL, respectively (Figure 5a). Under the more severe 70 °C conditions (Figure 5b), the free bacteriophages were completely inactivated (reducing by 7.56 ± 0.02 log PFU/mL), while the titer loss of the microencapsulated samples was significantly reduced (2.17 ± 0.04, 1.35 ± 0.02, and 1.41 ± 0.02 log PFU/mL, respectively). These results demonstrate that microencapsulation effectively protects bacteriophages from thermal inactivation, with multilayer coatings (CMCS2 and CMCS3) offering markedly better protection than the single layer. This approach offers a potential strategy for improving phage storage and application under high-temperature conditions. Overall, double-layer (CMCS2) and triple-layer (CMCS3) microcapsules offer a similar high level of thermal protection against bacteriophages. This phenomenon suggests that the protective efficacy of microcapsules against heat stress may stabilize after reaching a certain shell complexity.

### 3.6. Analysis of Phage Microcapsule pH Stability

**Figure 6:**
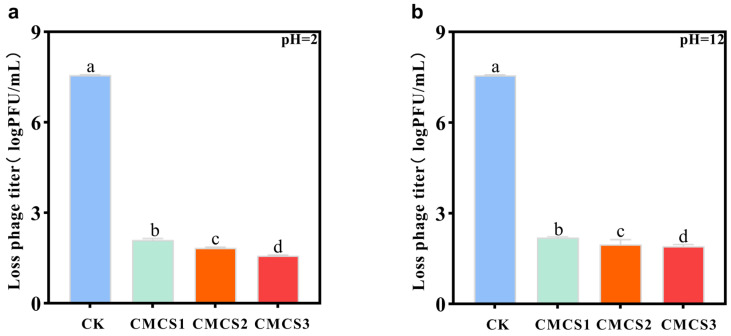
Titer loss of free phage and phage microcapsules with different layers under acidic and alkaline conditions. (**a**) pH 2; (**b**) pH 12. Error bars represent the standard deviation (SD) of three independent replicates.

To assess the effect of microencapsulation on phage pH tolerance, encapsulated phages were subjected to the same pH conditions as free phages. First, the titers of free phages in SM buffer at pH 2 and pH 12 for 1 h were determined, showing complete loss of activity in both cases. In contrast, microencapsulation markedly enhanced phage stability across pH extremes. Among the three microcapsule formulations, pH tolerance improved with increasing shell layers: CMCS1 < CMCS2 < CMCS3 (Figure 6). After 1 h at pH 2, titer reductions were 2.10 ± 0.05, 1.83 ± 0.02, and 1.59 ± 0.01 log PFU/mL for CMCS1, CMCS2, and CMCS3, respectively (Figure 6a). Under pH 12, the decreases were 2.20 ± 0.03, 1.97 ± 0.16, and 1.91 ± 0.07 log PFU/mL (Figure 6b). Although the underlying mechanism remains unclear, we speculate that changes in environmental pH may trigger gradual dissolution of the carboxymethyl chitosan–zein shell. This could delay phage exposure to harsh conditions while mitigating direct contact with extreme acidity or alkalinity, thereby improving phage survival. Unlike the results of thermal stability, under extreme pH stress, the protective efficacy of microcapsules shows a clear hierarchical dependence: CMCS3 > CMCS2 > CMCS1. This comparison indicates that there may be essential differences in the mechanisms by which multilayer shell structures resist thermal stress and pH stress.

### 3.7. The Stability of Microencapsulated Phages on Sea Bass Slices

**Figure 7:**
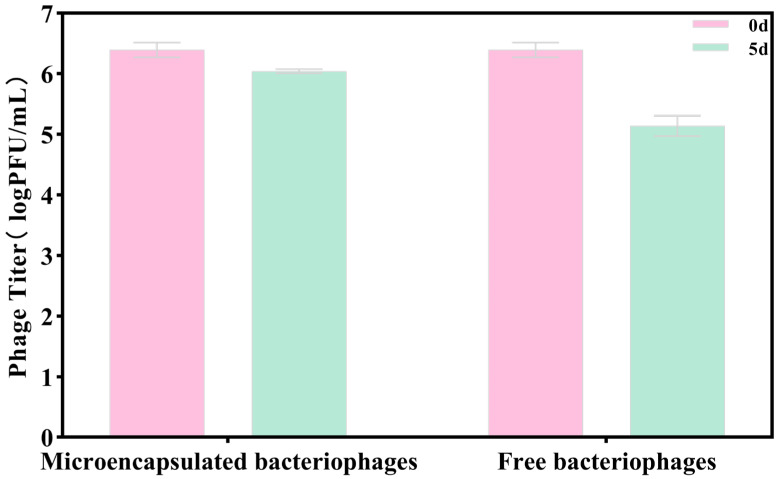
The titer changes of free phages and phage microcapsules on sea bass slices. Error bars represent the standard deviation (SD) of three independent replicates.

As shown in Figure 7, both microencapsulated and free bacteriophages maintained high stability on sea bass slices during 5 days of storage at 4 °C.

However, the two showed significant differences: compared with the free phage group, the microencapsulated phage group demonstrated higher stability. Specifically, after a 5-day storage period, the titer of the microencapsulated phage group decreased by only 0.35 log PFU/mL, while that of the free phage group decreased by 1.25 log PFU/mL. The main reasons for this stability difference may include the following points. Firstly, microcapsules provide effective physical protection for bacteriophages. Some bacteriophages are embedded in the microcapsule matrix, which protects them from the direct adverse environment on the fish surface during the initial storage period and enables their slow release, thereby delaying the decline in potency. Secondly, when free bacteriophages are directly exposed to the surface of fish slices, they are prone to uneven distribution or loss due to factors such as water migration and the exudation of the fish’s own juices, resulting in a rapid decrease in their effective concentration. Finally, there may be unfavorable factors such as proteases, lipid oxidation products, and microbial metabolic products on the surface of sea bass. Free bacteriophages may be more susceptible to the inhibitory effects of these factors and become inactivated. Ultimately, these factors collectively led to a more significant reduction in the titer of the free phage group.

### 3.8. The Release Behavior of Phage Microcapsules

**Figure 8:**
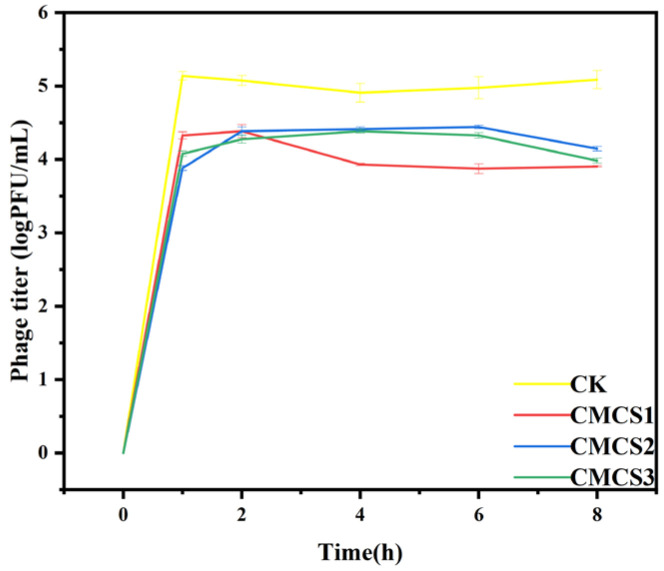
The release behavior of phage microcapsules. Error bars represent the standard deviation (SD) of three independent replicates.

To compare the release regulatory effects of different microcapsule formulations (CMCS1, CMCS2, CMCS3), we conducted an in vitro release study in a simulated fish exudate medium (PBS, pH 6.5, containing 0.3 M NaCl). This medium was designed to simulate the typical ionic strength and post-mortem pH fluctuations (generally ranging from 6.0 to 7.5) in sea bass muscle. The release profiles obtained under accelerated conditions (37 °C) are presented in Figure 8. All three types exhibited a rapid initial release within 60 min, reaching peak titers of 4.37 ± 0.07, 4.44 ± 0.02, and 4.39 ± 0.03 log PFU/mL, respectively, followed by a plateau phase that suggested a sustained release pattern. Notably, a decrease in titer was observed for the CMCS2 and CMCS3 groups after 8 h. We propose that two interrelated factors could explain this decline. First, the inherent instability of free phage particles in the release medium over extended periods could lead to a gradual loss of infectivity. Second, and more specific to the multilayer architecture, dynamic changes in the microcapsule wall material could promote a “re-adsorption” effect. During prolonged release, the polyelectrolyte walls of CMCS2 and CMCS3 could undergo partial swelling, erosion, or asymmetric degradation. This process exposed fresh charged fragments (positively charged zein residues or negatively charged CMCS chains), which could electrostatically interact with recently released phage particles, causing their transient re-adsorption or aggregation.

### 3.9. pH Analysis of Sea Bass Slices During Refrigeration Storage

**Figure 9:**
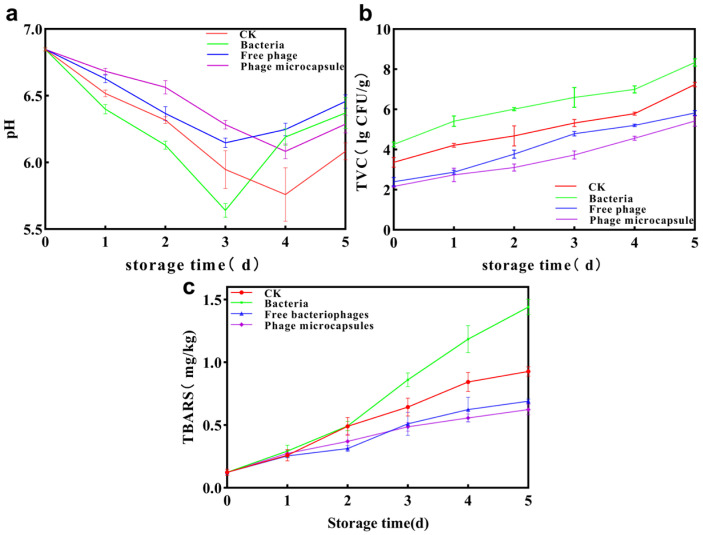
The pH (**a**), total viable count (TVC) (**b**) and thiobarbituric acid reactive substances (TBARS) (**c**) changes of sea bass slices during refrigeration storage. Error bars represent the standard deviation (SD) of three independent replicates.

Monitoring pH plays a pivotal role in assessing and ensuring the freshness and safety of aquatic products across the supply chain, from post-harvest handling to consumer purchase. Changes in the pH of sea bass during refrigerated storage under different treatment conditions are presented in Figure 9a. As shown, the pH values in all groups decreased continuously in the early storage stage. This decline may be attributed to the anaerobic glycolysis of glycogen in fish muscle tissue, leading to lactic acid formation, as well as the degradation of ATP into acidic compounds such as succinic acid. Consequently, the accumulation of these acidic metabolites resulted in a gradual reduction in pH. This declining trend, however, reversed in the later stage of storage, during which the pH increased rapidly. This rise can be explained by microbial growth and metabolism along with the enzymatic degradation of proteins by endogenous proteases, both of which generate alkaline substances, including amines, ammonia, trimethylamine, and other nitrogenous compounds [37,38,39]. The combined action of microorganisms and enzymes contributed to the increase in pH. By the 5th day of storage, the pH of the control group had reached 7.28 ± 0.07, whereas the pH in the phage-microcapsule treatment group was only 6.28 ± 0.07. These results demonstrate that the application of phage-based preservation significantly delayed the pH increase and retarded spoilage in sea bass, effectively extending its shelf life.

### 3.10. Total Viable Count (TVC) Analysis of Sea Bass Slices During Refrigeration Storage

Total viable count (TVC) serves as a fundamental and quantitative microbiological criterion for objectively assessing the freshness and evaluating the hygienic quality of fish products. A value exceeding 6 log (CFU/g) is generally considered to have reached the spoilage threshold [40]. This is mainly due to the autolysis of endogenous enzymes in meat products, which releases a large amount of nutrients, providing favorable conditions for the proliferation of microorganisms [41,42]. The experimental results are shown in Figure 9b. By the 4th day of storage, the TVC of the blank control group had rapidly risen to a level close to spoilage, while the TVC of the artificially contaminated group even reached 7.00 ± 0.17 log (CFU/g), significantly exceeding the spoilage threshold. In contrast, both phage treatment groups demonstrated significant preservative effects. Until the 5th day, the TVC of the phage microcapsule group and the free phage group remained at 5.42 ± 0.26 and 5.82 ± 0.12 log (CFU/g), respectively, and neither exceeded the spoilage threshold. This effective antibacterial effect mainly stems from the targeted lysis of specific FB06 artificially inoculated by bacteriophages. Although no significant difference in the antibacterial effect was observed between the two treatment groups (possibly related to the consistent initial titer of the bacteriophages used in the experiment), the results of this study clearly confirm that bacteriophages, as natural antibacterial agents, have good application potential in inhibiting specific spoilage bacteria in aquatic products and delaying quality deterioration.

### 3.11. TBARS Analysis of Sea Bass Slices During Refrigeration Storage

The content of thiobarbituric acid reactive substances (TBARS), principal secondary products of lipid oxidation, is a widely adopted metric for evaluating the extent of lipid oxidation in aquatic products during post-mortem storage [43]. In fish muscle, which is rich in polyunsaturated fatty acids, lipid oxidation proceeds via autocatalytic reactions initiated by oxygen, metal ions, or light. The resulting lipid hydroperoxides are subsequently decomposed into malondialdehyde (MDA), a recognized marker of spoilage in fish meat. Furthermore, MDA can induce protein cross-linking, while further oxidation generates volatile compounds such as alcohols, aldehydes, and ketones, which contribute to the development of off-odors [44]. The changes in TBARS values for the different sample groups throughout the storage period are illustrated in Figure 9c. The initial TBARS value of the sea bass samples was approximately 0.12 mg MDA/kg. Overall, the TBARS values of all groups exhibited a continuous increasing trend as storage time progressed, indicating ongoing lipid oxidation, albeit at slightly different rates among the groups. By the 5th day of storage, the TBARS value of the phage-treated fish meat was significantly lower than that of the contaminated control group. Specifically, the free phage group and the phage microcapsules group showed relatively slower increases in TBARS, reaching 0.69 mg/kg and 0.62 mg/kg, respectively, which were 0.75 mg/kg and 0.817 mg/kg lower than the value recorded for the contaminated group. Lower TBARS values in meat products generally indicate a lower degree of lipid oxidation and higher freshness. The observed difference was likely attributable, at least in part, to the targeted antibacterial activity of the bacteriophages, which may have delayed lipid oxidation in the fish meat by lysing spoilage bacteria, thereby extending the product’s shelf life. In addition, CMCS itself may also have contributed to a synergistic antibacterial effect.

### 3.12. (Total Volatile Basic Nitrogen) TVB-N Analysis of Sea Bass Slices During Refrigeration Storage

**Figure 10:**
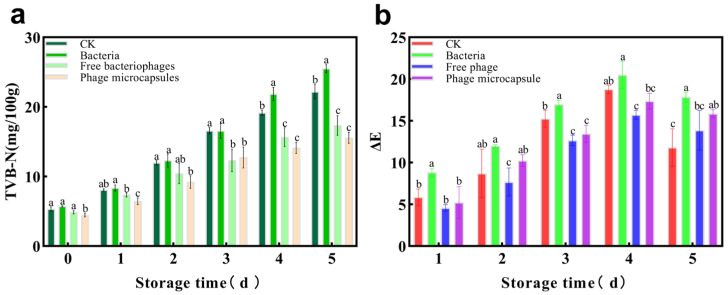
The (**a**) TVB-N (total volatile basic nitrogen) and (**b**) ΔE changes of sea bass slices during refrigeration storage. Error bars represent the standard deviation (SD) of three independent replicates.

During refrigeration storage, the increase in total volatile basic nitrogen (TVB-N) in fish meat is primarily attributed to microbial proliferation and metabolism, which degrade proteins into ammonia and amines [45]. As shown in Figure 10a, the initial TVB-N value of sea bass was 5.34 ± 0.29 mg/100 g. Throughout storage, TVB-N increased in all groups. In the control group, the value reached 22.19 ± 1.0 mg/100 g by day 5, exceeding the acceptability limit of 20 mg/100 g. In contrast, TVB-N in the two treatment groups remained below this threshold, owing to effective suppression of specific spoilage bacteria by bacteriophage treatment. By inhibiting the growth of putrefying bacteria, phage application delayed microbial-mediated protein degradation and enzymatic reactions, thereby slowing TVB-N accumulation and extending the shelf life of fish. These results demonstrate that TVB-N is not only a reliable indicator of freshness but also reflects the efficacy of preservation strategies in controlling microbial and biochemical deterioration.

### 3.13. Color Analysis of Sea Bass Slices During Refrigeration Storage

The color of meat products is a critical factor affecting consumer preference. In this study, color parameters including lightness (L), redness (a), yellowness (b), and the total color difference (ΔE) were measured to evaluate the color changes in sea bass slices during storage. The values of L*, a*, and b* are presented in Table 2, Table 3, and Table 4, respectively, while the changes in ΔE are shown in Figure 10b.

During storage, L* values gradually decreased, while b* values increased across all groups, indicating a consistent trend of surface darkening and yellowing. The change in the a* value followed distinct patterns depending on the treatment, with a pronounced and continuous decrease observed only in the free phage group, while values in the other groups fluctuated over time. These color shifts, reflected in the rising ΔE values, not only compromised visual appearance but also indicated a decline in freshness and nutritional quality. Mechanistically, the decrease in L* values is primarily attributed to the oxidation of myoglobin to metmyoglobin [46], while the increase in b* values is associated with the accumulation of lipid oxidation products [47,48] and microbial metabolic activity [49]. Notably, phage-based treatments—especially the microcapsule group—showed a significantly smaller reduction in L* values and a lower overall ΔE increase compared to the controls, corresponding to their better microbial and oxidative stability. These results suggest that phage treatment delayed color deterioration by suppressing spoilage bacteria and their associated oxidative pathways, supporting the potential of bacteriophages as promising biopreservatives for seafood.

## 4. Conclusions and Discussion

This study successfully developed a novel bacteriophage microcapsule system via layer-by-layer self-assembly using food-grade zein and CMCS. The spectroscopic data provide insights into the interaction mechanisms. The downfield shift of the amide I band in FT-IR is consistent with electrostatic interaction between zein and CMCS, while the shift in the O–H stretching region suggests the likely involvement of hydrogen bonding. Together with the alternating zeta potential profile, these results strongly support that electrostatic attraction is the primary driving force for the LbL assembly, with hydrogen bonding potentially contributing to the stability of the multilayered structure. Furthermore, the absence of new protein bands in SDS-PAGE indicates that covalent cross-linking was not a major event under the current preparation conditions. Encapsulation preserved phage viability, with only moderate titer loss. Compared to free phages, microencapsulated phages exhibited markedly improved thermal stability (60 °C and 70 °C) and pH tolerance (pH 2.0 and pH 12.0). Release studies in simulated fish exudate showed rapid initial release followed by sustained delivery, suggesting immediate and prolonged antibacterial action.

In the preservation test based on a specific model (lamb fish slices inoculated with the specific host bacterium *Acinetobacter junii* FB06), the drug-loaded microcapsules demonstrated the potential to inhibit the growth of the target bacteria. Specifically, compared with the control group without inoculation, the total colony count, volatile basic nitrogen, and thiobarbituric acid reaction product values in the treated group rose more slowly, the pH value increase during refrigeration was delayed, and the color stability was better maintained under the test conditions. These results collectively indicate that the zein/CMCS layer-by-layer assembly platform has application potential in delivering bacteriophages in food matrices. Further studies should focus on process scaling-up, evaluate the performance of this platform in combating complex natural spoilage bacterial communities in various real seafood matrices, and use advanced imaging and simulation techniques to elucidate the protective mechanism of the multilayer structure.

This study reveals that the protective efficacy and mechanism of microcapsules evolve with the increase in the number of layers. The single-layer structure only provides basic isolation, while the double-layer structure (CMCS2) builds a dense interface with both thermal stability and certain ion buffering capacity through the combination of hydrophobic zein and hydrophilic CMCS, achieving initial functional synergy. The key breakthrough emerged in the three-tier structure (CMCS3), whose protective mechanism exhibits significant stress specificity.

For heat stress, the protective effect did not significantly improve on the double-layer basis, indicating that its heat barrier capacity may have approached a plateau. This observation is consistent with the “saturation effect” in thermal insulation for multilayer structures, where increasing the number of layers beyond a certain point yields diminishing returns in protective efficacy. This phenomenon has also been noted in other polyelectrolyte multilayer systems and in LbL microcapsules with similar protein–polysaccharide architecture [28].

For pH stress, the protective effect continuously increases with the number of layers. This is attributed to the dynamic response mechanism of the multilayer polyelectrolyte shell under extreme acids and bases: the outer material is the first to consume or buffer a large number of H^+^/OH^−^ ions through swelling, charge neutralization and other means, thereby delaying the exposure and deactivation time of the internal core and achieving sequential protection from the outside to the inside.

The primary focus and contribution of this work lie in the development and validation of an LbL microcapsule platform for phage delivery. This platform has been effectively demonstrated using the model phage Zk-X6 and its specific host, showing excellent performance in enhancing environmental tolerance and controlling release in vitro and in a simplified food model. It is important to emphasize that this constitutes a proof-of-concept study. To translate this platform into a practical biocontrol solution, future work must integrate it with phages subjected to comprehensive biological characterization. This includes a thorough safety and efficacy assessment encompassing phage host range, one-step growth kinetics, frequency of resistance development, and potential impact on the human gut microbiota.

## Figures and Tables

**Table 1:** Phage titer recovered from ruptured microcapsules and the corresponding encapsulation efficiency.

Sample	Free State	CMCS1	CMCS2	CMCS3
Phage titer	5.14 ± 0.01 ^a^	4.05 ± 0.04 ^b^	4 ± 0.07 ^bc^	3.94 ± 0.06 ^cd^

Note: Different superscript letters within the same row denote statistically significant differences (*p* < 0.05). Phage microcapsules with 1, 2, and 3 encapsulation layers are abbreviated as CMCS1, CMCS2, and CMCS3. The encapsulation efficiency (*EE*) values were calculated from the mean logarithmic titers shown above after conversion to linear units (PFU/mL), as per Equation (1).

**Table 2:** The L* value change of sea bass slices during refrigeration storage.

Time (*d*)	CK	Bacteria	Free Phage	Phage Microcapsules
0	53.97 ± 0.83 ^a^	55.17 ± 0.45 ^a^	55.17 ± 0.45 ^a^	54.78 ± 1.2 ^a^
1	48.35 ± 0.64 ^b^	46.52 ± 0.49 ^c^	50.79 ± 0.71 ^a^	49.68 ± 1.12 ^ab^
2	45.86 ± 2.68 ^ab^	43.73 ± 0.17 ^b^	47.8 ± 1.38 ^a^	45.04 ± 0.59 ^ab^
3	40.54 ± 1.63 ^b^	39.26 ± 0.58 ^b^	42.86 ± 0.25 ^a^	42.82 ± 1.35 ^a^
4	37.14 ± 0.66 ^bc^	35.58 ± 1.63 ^c^	40.43 ± 0.41 ^a^	38.21 ± 0.5 ^b^
5	44.47 ± 2.41 ^a^	38.18 ± 0.78 ^b^	42.03 ± 2.66 ^ab^	39.88 ± 1.06 ^b^

Note: The significance level was set at α = 0.05. For the presentation of results, data in the tables are shown as mean ± SD (*n* = 3). Within the same storage time point, mean values bearing different superscript letters indicate statistically significant differences between treatment groups (*p* < 0.05), whereas the same letter denotes no significant difference.

**Table 3:** The a* value change of sea bass slices during refrigeration storage.

Time (*d*)	CK	Bacteria	Free Phage	Phage Microcapsules
0	−4.22 ± 0.2 ^c^	−3.4 ± 0.33 ^b^	2.46 ± 0.09 ^a^	−3.44 ± 0.11 ^b^
1	−3.11 ± 0.22 ^b^	−2.9 ± 0.22 ^b^	1.7 ± 0.47 ^a^	−2.81 ± 0.17 ^b^
2	−1.77 ± 0.39 ^b^	−0.98 ± 0.41 ^b^	1.3 ± 0.4 ^a^	−0.94 ± 0.44 ^b^
3	−1.75 ± 1.15 ^b^	−2.6 ± 0.25 ^b^	0.82 ± 0.25 ^a^	−2.7 ± 0.09 ^b^
4	−1.95 ± 0.6 ^a^	−2.45 ± 0.65 ^a^	−2.24 ± 1.15 ^a^	−0.88 ± 0.49 ^a^
5	−2.34 ± 0.42 ^b^	−1.81 ± 0.88 ^ab^	−1.13 ± 0.13 ^a^	−1.06 ± 0.46 ^a^

Note: The significance level was set at α = 0.05. For the presentation of results, data in the tables are shown as mean ± SD (*n* = 3). Within the same storage time point, mean values bearing different superscript letters indicate statistically significant differences between treatment groups (*p* < 0.05), whereas the same letter denotes no significant difference.

**Table 4:** The b* value change of sea bass slices during refrigeration storage.

Time (*d*)	CK	Bacteria	Free Phage	Phage Microcapsules
0	−1.72 ± 0.62 ^a^	−1.07 ± 0.46 ^a^	−0.92 ± 0.52 ^a^	−1.33 ± 0.43 ^a^
1	−0.65 ± 0.42 ^a^	0.28 ± 0.84 ^a^	−0.01 ± 0.22 ^a^	−0.61 ± 0.41 ^a^
2	−0.07 ± 0.13 ^b^	1.51 ± 0.36 ^a^	0.76 ± 0.63 ^ab^	0.43 ± 0.5 ^b^
3	−0.57 ± 0.7 ^c^	2.12 ± 0.1 ^a^	1.47 ± 0.53 ^ab^	0.15 ± 1.01 ^bc^
4	1.85 ± 0.83 ^ab^	2.43 ± 0.81 ^a^	1.68 ± 0.27 ^ab^	0.16 ± 1.15 ^b^
5	0.35 ± 0.89 ^b^	2.26 ± 1.13 ^a^	1.48 ± 0.08 ^ab^	1.12 ± 0.57 ^ab^

Note: The significance level was set at α = 0.05. For the presentation of results, data in the tables are shown as mean ± SD (*n* = 3). Within the same storage time point, mean values bearing different superscript letters indicate statistically significant differences between treatment groups (*p* < 0.05), whereas the same letter denotes no significant difference.

## Data Availability

The original contributions presented in this study are included in the article. Further inquiries can be directed at the corresponding author.

## Supplementary Materials

The following supporting information can be downloaded at: https://www.mdpi.com/article/10.3390/foods15061032/s1, Figure S1: Phylogenetic tree (Development tree) of the bacterial strains; Figure S2: Sequence alignment and accession number identification; Data S1: Original 16S rDNA sequence file (FB06_16S_C10772).

## Abbreviations

CMCS	Carboxymethyl Chitosan
LbL	Layer-by-Layer
TVC	Total Viable Count
TBARS	Thiobarbituric Acid Reactive Substances
TVB-N	Total Volatile Basic Nitrogen
PBS	Phosphate-Buffered Saline
