# Supplementary figures and images for: Development of a Layer-by-Layer Zein/CMCS Microcapsule Platform for Bacteriophage Delivery: A Proof-of-Concept Study Using a Model Phage in Sea Bass
Source: Foods. 2026 Mar 16;15(6):1032. doi: 10.3390/foods15061032 (PMC13024753; doi:10.3390/foods15061032)

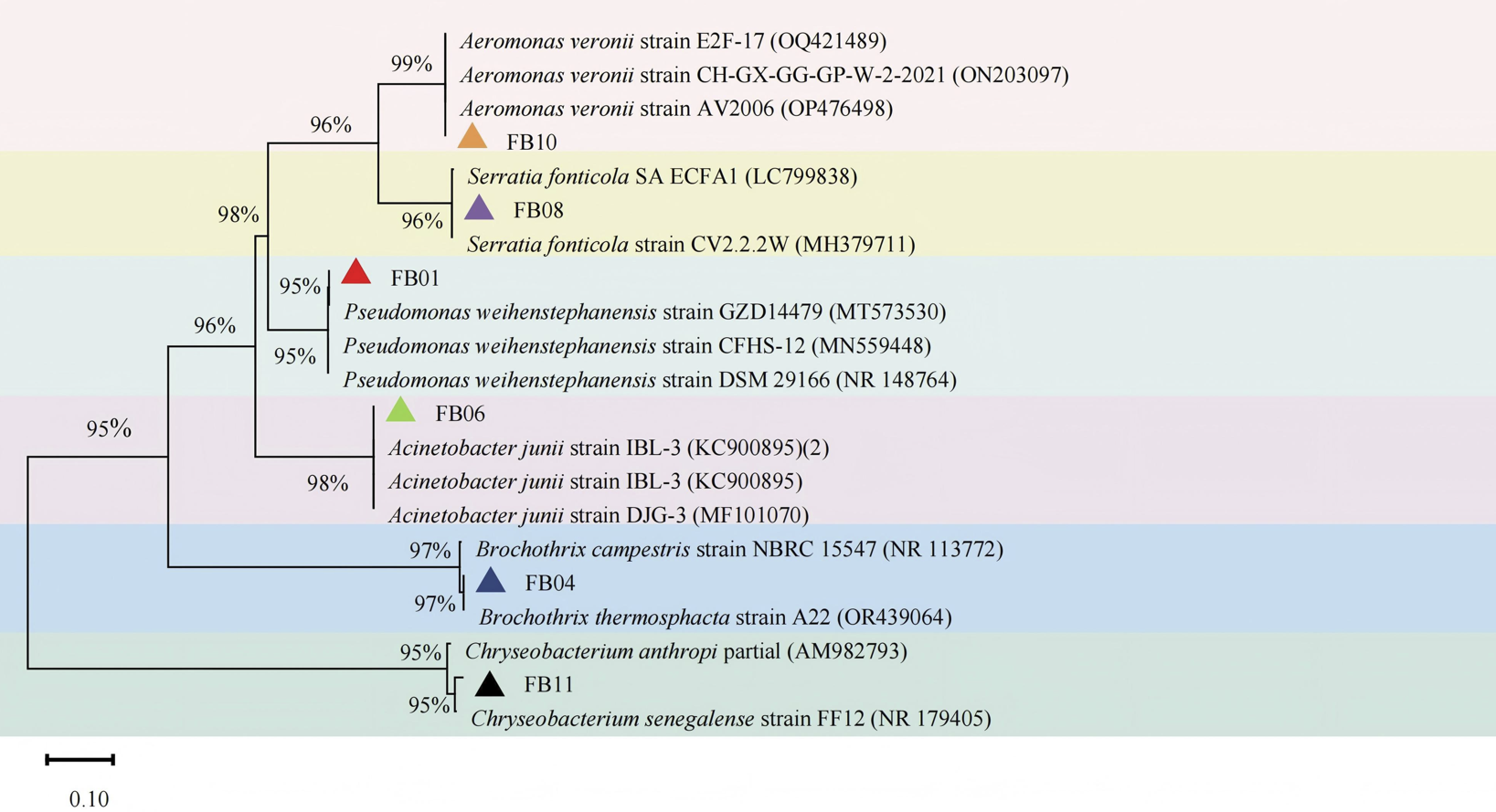

Supplement: Supplementary file 1 [file foods-15-01032-s001.zip › Figure S1 Phylogenetic tree (Development tree) of the bacterial strains.png]

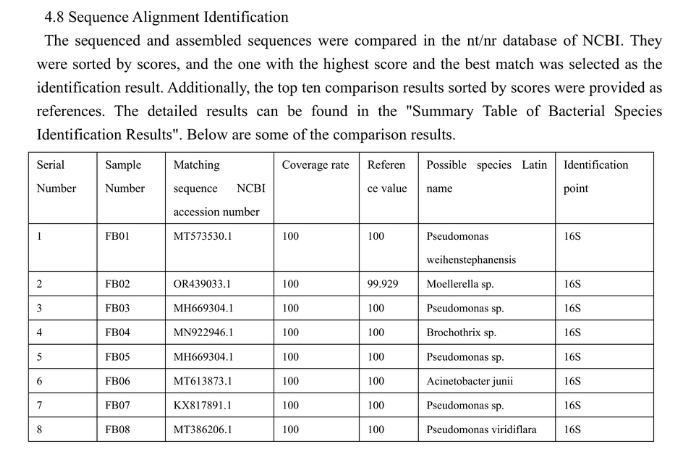

Supplement: Supplementary file 1 [file foods-15-01032-s001.zip › Figure S2 Sequence alignment and accession number identification .png]
